# Increasing densities of *Leucosidea sericea* have minimal effects on grazing capacity and soil characteristics of a high-altitude communal rangeland at Vuvu, South Africa

**DOI:** 10.1371/journal.pone.0308472

**Published:** 2024-09-06

**Authors:** Nandipha Gloria Ndamane, Manqhai Kraai, Ntuthuko Raphael Mkhize, Tlou Julius Tjelele, Zivanai Tsvuura

**Affiliations:** 1 Centre for Functional Biodiversity, School of Life Sciences, University of KwaZulu-Natal, Scottsville, South Africa; 2 Department of Biological and Agricultural Sciences, School of Natural and Applied Sciences, Sol Plaatje University, Kimberley, South Africa; 3 Agricultural Research Council, Animal Production, Range and Forage Sciences, Irene, South Africa; Museu Paraense Emilio Goeldi, BRAZIL

## Abstract

Increasing densities of woody plants, known as woody plant encroachment, is a phenomenon affecting savannas and grasslands in many parts of the world. Yet, these ecosystems sustain a significant proportion of the human population through the provision of ecosystem services, such as forage for livestock and wildlife production. While low to medium altitude rangelands are encroached by many species of woody plants, high altitude rangelands in southern Africa show increasing densities of *Leucosidea sericea*, a woody shrub or small to medium-sized tree. Influences of this species on rangeland dynamics are unknown. This study aimed to determine the influence of *L*. *sericea* on rangeland functioning in the Vuvu communal area in the Eastern Cape, South Africa. Effects of *L*. *sericea* on plant species diversity and composition, rangeland condition and grazing capacity were measured in sites of variable densities of the species in topographical locations designated as plains, upland and stream sites, using a point-to-tuft method along 50-m long transects. Soil samples were collected to a depth of 5 cm from plains, streams, and upland sites, and analysed for organic carbon, nitrogen, phosphorus, magnesium, calcium, and pH. Plant species richness and abundance were similar among topographical locations, which was reflected by the similar Shannon-Weiner (*H′*) diversity indices among sites. Topographical locations differed significantly in species composition. The plains sites had a higher grazing capacity than stream sites, which had a grazing capacity similar to that of upland sites. Values of soil physicochemical properties were similar among the sites. Overall, soils were acidic (range in pH: 4.4–4.6) and had low amounts of organic carbon and total nitrogen. These findings suggest that *L*. *sericea* is not the primary cause of rangeland degradation as all sites were in poor condition as shown by the low grazing capacity, poor rangeland condition and depauperate species richness and diversity. Therefore, rangeland management should shift towards restoration strategies aimed to revitalise the rangeland.

## Introduction

Increasing densities of woody plants in grasslands and savannas is a well-known and globally widespread phenomenon, called woody, or bush encroachment [1–3]. In the most heavily encroached rangelands, a single woody plant species can cover up to 75% of the ground surface, resulting in impenetrable thickets that limit the establishment of grasses, and negatively impact grazing [4–6]. It is not only the increasing density of woody plants that is of major concern, but also the rate at which it occurs [7]. For example, a study that analysed aerial photographs from 1940 to 2010 indicated that woody cover had increased in commercial, communal and conservation areas in South Africa [8]. A local scale study based on repeat, fixed point photography showed that woody plant cover had increased in KwaZulu-Natal over the last 200 years, and the increase was attributed to, among others, increased grazing pressure, reduced fires and reduced use of firewood as a result of greater access to electricity [9]. Encroachment by woody plants has been linked to changes in ecosystem structure and function [10], and the reason for the conversion of grasslands to woody dominated ecosystems is still a subject of controversy. While some researchers argue that the alterations are natural or human induced [11], others [12, 13] have concluded that a combination of both is responsible for bush encroachment. Among other factors, land abandonment, overgrazing, exclusion of browsing species, altered fire frequency, climate change, and global warming have all been linked to woody encroachment, especially in the grasslands and savannas of Africa [13, 14].

The loss of herbaceous plants (grasses and forbs) is one of the most concerning aspects of the increase in woody vegetation density on rangelands [15]. Moreover, increased cover of woody plants can directly affect herbaceous layers by altering grass and forb diversity and composition [16]. At high levels, woody plant cover may lead to a loss of annual and perennial grasses which are major forage resources to grazers [17], threatening livestock production [18]. In East Africa, rangelands have lost 40–80% of highly palatable forage grasses as a result of increased woody plant cover [15].

Woody plant encroachment may contribute to alterations in soil chemical properties, either decreasing or increasing amounts of soil chemicals, however, these impacts on soil properties are less understood [19]. Improved understanding of the interaction of woody plants and their environment is necessary for better management and conservation of rangeland ecosystems [20, 21]. Literature provides evidence suggesting that changes in land-use practices, coupled with overgrazing have largely contributed to increased woody plant density, which in turn influence soil carbon sink and soil nitrogen availability [14, 22, 23]. In particular, [24] showed that increasing tree density and cover along a woody plant encroachment gradient led to increased carbon (C), nitrogen (N) and phosphorous (P) in the shallow plinthic soil’s uppermost layer. Additionally, [25] reported that basal respiration and soil microbial biomass were greater in encroached than non-encroached mesic sites in South Africa. Their results were attributed to larger amounts of soil organic carbon and total N in encroached sites, which was driven by greater plant biomass in encroached sites. However, these results were not consistent across the precipitation gradient that was studied and where the encroaching species were nitrogen-fixing legumes. Consequently, there is limited knowledge of the extent to which major soil nutrients are affected by vegetation changes such as increased tree density in grasslands and savannas.

*Leucosidea sericea* Eckl. & Zeyh. (Rosaceae) is a problematic encroaching woody plant species occurring in the highlands of southern Africa [26]. The plant grows into dense thickets on overgrazed areas at elevations above 1000 m, near water, in grassland and on mountain slopes [26, 27]. Despite the tendency for *L*. *sericea* to become a dominant woody plant in eastern highlands of southern Africa, little is known about the impact of its proliferation on rangeland condition, plant species composition (e.g. grasses, woody plants and forbs), and grazing capacity of communally-managed rangelands. Given that livestock production in the eastern parts of South Africa is based predominantly on communal rangelands, knowledge on the impacts of *L*. *sericea* encroachment is crucial for management of these rangelands.

We investigated impacts of the dominant woody plant, *L*. *sericea*, on rangeland condition in the Vuvu communal area in the Eastern Cape Province of South Africa. Pastoralists in these areas regard their livestock as being a “living bank”, or a source of money and prestige, which establishes one’s social standing [28, 29]. While livestock is a major asset in Vuvu, pastoralists also derive other uses and benefits from *L*. *sericea*, such as firewood, durable fence posts and medicines [30]. To our knowledge, effects of this species on grazing capacity, plant diversity and veld condition are largely unknown. However, *L*. *sericea* has prolific coppicing ability after fire, causing high-density patches of the species when fires are infrequent, and low-density patches when fires are frequent [31].

The aim of this study was to assess how *L*. *sericea* density influences grazing capacity, species diversity of herbaceous plants and veld condition of a high-altitude rangeland at Vuvu in the Eastern Cape Province of South Africa. The Vuvu area lies on the south-eastern boundary of the Drakensberg Mountain Centre of plant endemism, which consists of endemics distributed in a montane and an alpine sub-centre [32]. The conservation value of the area will be further enhanced by the mooted establishment of the North East Cape Grasslands National Park. To determine the impact of *L*. *sericea* encroachment on rangeland condition, we selected three categories of sites consisting of plains, stream sites, and uplands. The plains are open-grassland sites where *L*. *sericea* occurs at low density while stream sites are close to the main drainage channels and have significant densities of *L*. *sericea*. The uplands are mountain slopes which also showed the presence of *L*. *sericea* plants. Because of greater densities of *L*. *sericea* plants in uplands and streams, we assumed that grazing capacity and soil nutrient content would be driven by *L*. *sericea* compared to the plains. Therefore, we hypothesised that herbaceous plant species richness, species composition, basal cover, veld condition and grazing capacity would be lower in uplands and along streams than the plains.

## Materials and methods

### Study area and species

The study was conducted at Vuvu communal area (30.6914°S, 28.5051°E) located in Joe Gqabi District, Eastern Cape Province, South Africa. The area is part of the southern Drakensberg highlands with vegetation described as East Griqualand Grassland that occurs at high altitude (1720–1900 m.a.s.l.) where dense tussock grasses dominate [33]. The vegetation is dominated by grass species such as *Aristida junciformis*, *Heteropogon contortus*, *Eragrostis curvula*, *Sporobolus africanus* and *Hyparrhenia hirta* [27]. Native woody plants include *Buddleja salviifolia*, *Diospyros lycioides*, and *Leucosidea sericea* including alien timber trees such as *Eucalyptus* spp. and *Acacia mearnsii*.

Soils are derived from basalts of the Andisols and mudstones of the Mollisols [34]. Shale and mudstone of the Mollisols and Molteno Formations dominate the catchment lithology at low altitude while basalts of the Andisols dominate at high altitude [34]. Smaller areas of sandstones (Entisols) separate these two lithologies and soils are heavily leached. Entisols are often shallow, well drained, and may have low nutrient holding capacity. Soils developed from the Mollisols shales and mudstones are dispersive and highly erodible [35]. Soils developed from Entisols are less erodible, but steep slopes increase the erosion potential.

The mean annual rainfall of 635 mm falls mainly from October to March. Mean minimum and maximum monthly temperatures are 10.5ºC in July and 31.4ºC in February, respectively, with frost and snow common at higher elevations in June and July [33].

*Leucosidea sericea* is a small shrub to medium-sized evergreen tree which can be single or multi-stemmed. The species is frost resistant, which is an important adaptive trait to have given its occurrence at high altitudes. In South Africa, *L*. *sericea* occurs in all provinces except the Western Cape and the Northern Cape, and its distribution extends north into Namibia and central Zimbabwe [36]. *Leucosidea sericea* is a resprouter that uses underground carbohydrate reserves to initiate coppice regrowth following topkill by fire [31]. Plant regeneration by resprouting suggests that plant establishment from seed is poor [37, 38].

Poor agricultural practices and the steep topography has resulted in accelerated soil erosion, which is evident as significant sheet and rill erosion with deep gullies are visible in places [39]. The mass roll-out of social grants from the late 1990s reduced reliance on subsistence crop farming and land use shifted to predominantly grazing so that many areas formerly used for crop production are now fallow [40]. Abandonment of agricultural land is not only evident in many parts of the country, but a global phenomenon [41]. At the same time, the number of all grazing animals (e.g. cattle, sheep, goats, and horses and donkeys) in the area has increased markedly since the early- to mid-2000s (Eastern Cape Provincial Department of Agriculture, Extension and Advisory Services, Unpublished data) although the area has been grazed for more than 100 years [39].

### Ethical considerations and permission to undertake the research

This research was undertaken under the auspices of the Agricultural Research Council and the University of KwaZulu-Natal. When this research was carried out, neither organisation required ethical approval for field studies that did not involve vertebrate subjects. Permission to undertake research in Vuvu was obtained from the traditional authority of the area, led by the headman, Mr Christopher Nkosana Siphambo. Granting of permission was preceded by a community meeting at which the research team (NGN, MK, NRM, ZT) presented the research idea to the community. Input from community members led to the refinement of the overall research from which this paper forms part.

### Site selection and experimental layout

We used three herders from the area to assist with identification and delimitation of the Vuvu rangeland. From this we identified three broad topographical locations, which we designated the plains (i.e. where *L*. *sericea* plants were absent) and the uplands and stream terraces where *L*. *sericea* occurs at high density. Uplands were defined as high elevation plateaux on top of the mountains at an altitude of ca. 1900 m with average slope of 8–13%. Streams were considered the steeper locations on the mountainsides and were ca. 1700 m with slope of 18–20% while plains were ca. 1600 m with slope of 14–16.7%. These locations were chosen to represent the prevailing state of the rangeland in the study area.

We collected baseline information on woody plant density and height at each of the topographical units using the point-centred quarter (PCQ) method [42]. The mean distance between trees on streams and upland sites was 5.24 and 4.83 m, respectively, which translates to mean densities of 7518 trees ha^-1^ in uplands and 6312 trees ha^-1^ in streams. Tree density between uplands and streams was similar (*F =* 0.33, df *=* 14, *P* > 0.05), but the designation as separate topographical units was retained because the topographical units could be different in soil characteristics and other vegetation attributes besides tree density. Short trees (< 1 m tall) contributed 68% and 49% to the density of *L*. *sericea* at stream and upland sites, respectively, while there were fewer trees in height categories of 1–2 m and > 2 m (S1 Fig). We established 15 sampling sites for each of the open grassland plains, streams and upland areas. Sites were at least 100 m apart, and at least 300 m from the nearest homesteads.

### Herbaceous vegetation sampling

At each site, we used a 50-m transect to collect data on herbaceous species richness, composition, and basal cover using a point-to-tuft method [43]. This method has been used to inventory herbaceous cover relative to soil type, woody vegetation cover (such as trees and shrubs), aspect and slope [43, 44]. To determine the tuft diameter for estimation of basal cover, the longest and shortest axis of grass was measured along the transect [45]. A thin wire rod was placed vertically to the ground at 1-m intervals along each transect. If the rod struck bare ground, the distance between the point on bare ground and nearest plant species was measured and recorded. When only the centre of the tuft was dead and the outer parts were alive, the entire tuft diameter was measured. If the rod hit bare ground greater than 40 cm diameter or width, the area was recorded as bare ground. Grasses were categorised into groups of ecological status such as decreaser, increaser I, increaser II, and increaser III, based on whether the grass species responds to grazing by increasing or decreasing in abundance [46]. In particular, [46] describes decreasers as those species that have high grazing value but decrease in abundance as the rangeland deteriorates under high grazing pressure and trampling. Increaser I species are generally less palatable climax grasses that are abundant in underutilised rangeland and under conditions of little or no herbivory. Increaser II species are those that increase with light to moderate grazing, while increaser III species are common in overgrazed rangeland where they increase in abundance with light to severe grazing pressure.

Rangeland condition was determined using ecological status and grazing values of the plants identified during the vegetation surveys. We used [47] for rangeland condition and ecological groupings, and [46] for grazing values. Forbs, shrubs and trees (if present) were also identified, however, non-grass herbaceous species that did not fall in the above categories (ecological status) were listed as ‘other’. Species richness, species composition, basal cover and grazing capacity were computed for each site category.

### Soil sampling and analysis

We collected three soil samples from each of the plains, streams and upland sites. Each soil sampling location was a 5 m × 5 m plot from which we used three quadrats (0.25 m × 0.25 m) located at two diagonal corners and the centre of each plot to collect the soil to a depth of 5 cm, taking care to collect from between- rather than underneath shrub canopies. The three soil samples from each plot were mixed to form one composite sample which was placed in plastic bags pending analysis in the lab. Thereafter, soils were air-dried, sieved through a 2 mm sieve before analysis for soil pH, exchangeable bases, exchangeable acidity, total C and total N. All physicochemical analysis was carried out by the Soil Fertility and Analytical Services Unit at Cedara, using standard methods as described in [48].

Soil pH was determined in 1M KCl using a 1: 2.5 soil to KCl suspension. A 10 g soil sample was added to 25 ml of 1M KCl. The mixture was stirred for 5 min at 400 r.p.m. Thereafter, a pH reading was measured using a pH meter. Exchangeable bases (Ca^2+^ and Mg^2+^) and acidity were extracted using 25 ml 1M KCl solution after shaking for 10 min in a shaker at a speed of 400 r.p.m. The concentrations of Ca^2+^ and Mg^2+^ were then determined using atomic absorption spectrophotometry. From the same extract, exchangeable acidity was determined using the acid-base titration. Potassium was extracted using the Ambic-2 method, followed by quantification using atomic absorption spectrophotometry. The total N and C content of the soil were determined by the automated Dumas dry combustion method using a LECO Trumac CN/NS analyser (LECO Corporation, St Joseph, MI, USA). The clay content of the soil was estimated by the mid-infrared reflectance using air-dried, milled samples.

### Data analysis

Species richness and abundance of plants in the transects were tested for normality using the Kolmogorov—Sminorv test while the assumption of homogeneity of variances among groups was tested using the Levene’s test. For each dependent variable, no transformation could normalise residuals of the data or homogenise variances and so the data were compared among site locations using the Kruskal-Wallis test. Plant species diversity at each site was calculated using Shannon-Wiener (*H*′) and Simpson (*D*)’s indices of diversity, while the evenness index was calculated as the ratio of the Shannon-Wiener index to the natural logarithm of number of species in the sample [49]. The Shannon-Wiener index is an information statistic which assumes all species are represented in a sample and that they are randomly sampled, while the Simpson’s index is based on dominance because it gives more weight to common or dominant species [50]. We used both indices of diversity to explore rarity and dominance patterns of species at sites and among site categories. Plant species diversity was tested for normality and homogeneity of variances, then compared among the site categories using one-factor analysis of variance (ANOVA).

To determine species composition and abundance among site categories, we used non-metric multidimensional scaling (NMDS) ordination in R Vegan: Community Ecology package [51]. The NMDS was generated from the Bray-Curtis dissimilarity matrix based on whether species were shared among site categories. Sites that were close together in the NMDS ordination space were considered similar in species composition, while sites that were far apart were interpreted as containing different species. Patterns of species similarity shown in the NMDS were confirmed using analysis of similarity (ANOSIM). ANOSIM gives a probability value and a statistic R, which indicates the strength of the factors on the samples. Values of R range from 0 to 1. An R value close to 1 suggests dissimilarity between sites while an R value close to 0 suggests an even distribution [52]. Similarity percentages (SIMPER) were calculated using Past 4.03 [53] to determine the contribution of each species to dissimilarity among site categories.

We calculated basal cover for each sampling point along a transect using the equation by [45]:

$$\text{BC=}19.8+0.39(D)-11.87({\text{log}}_{\text{e}}D)+0.64(d)+2.93({\text{log}}_{\text{e}}d)$$

where BC is basal cover, *D* is the mean distance (cm) from a point to the nearest tuft, and *d* is the mean basal diameter (cm) of the tuft. Basal cover was arcsine square-root transformed to homogenise variances before comparison among site categories and ecological groups using generalised linear models, and the best goodness of fit was realised with a normal distribution with identity link function.

Rangeland condition scores per species were calculated as a product of the number of times a species occurs in a transect and its forage factor (also called grazing value), which is an index of forage production potential for each species. Forage factors range from zero (low potential for grazing) to ten (high potential for grazing) [43]. For the purposes of this study, forage factors were only presented for the grasses. Rangeland condition scores of the species were not normally distributed and so were compared among topographical locations using the Welch ANOVA followed by the Games-Howell *post hoc* test.

We measured grazing capacity following [54]:

$$\text{GC}=\{(-0.03+0.00289\times (X_{1})+[(X_{2}-419.7)(0.000633)]\}$$

where GC = grazing capacity in large stock units per hectare, *X*_1_ = percentage veld condition score, *X*_2_ = mean annual rainfall in mm. The grazing capacity estimates were calculated by substituting into the equation the veld condition score as a percentage and the mean annual rainfall for Vuvu for 1994 to 2018 of 661 mm. Grazing capacity was tested for normality before comparing among site categories using ANOVA. Where significant, ANOVA was followed by the Tukey post hoc test for pairwise comparisons of group means.

Soil chemical properties (e.g. amounts of N, P, K, Na, Mg) were tested for normality before comparing among site categories using multivariate analysis of variance (MANOVA). Normality tests showed that values of exchangeable acidity, acid saturation, K, Cu and clay content were not normally distributed. A square-root (sqrt[x+0.25]) and log_10_(x+1) transformation [55] restored normality to the exchangeable acidity and Cu data, respectively, while no suitable transformation resulted in normality of acid saturation, clay and K. We then carried out bivariate correlation tests among the soil variables to determine strongly correlated (*r* > 0.96) variables whose inclusion in the MANOVA model would lead to redundancy and collinearity [56]. Acid saturation, total cations and total N were removed from the model as they were strongly correlated to exchangeable acidity, Mg and organic C, respectively. Homogeneity of variance tests showed that all variables included in the MANOVA met the assumption of equal variances among groups for each variable. In all statistical analysis, we used a significance level of α = 0.05.

## Results

### Plant species diversity

A total of 32 plant species were recorded, which consisted of grasses (66%), forbs (22%) and woody plants (12%). A total of 22 herbaceous species were recorded in the plains. The 19 plant species recorded in streams included *Leucosidea sericea* and *Diospyros lycioides* among the woody plants, while the 16 species in uplands also included *L*. *sericea* in relatively high abundance. However, the number of plant species was similar among the plains, streams and uplands (Kruskal-Wallis *H* = 3.247, df = 2, *P* = 0.197). At all sites the most commonly found grasses were in the increaser II category (e.g. *Eragrostis curvula*, *E*. *plana*) which are less palatable. The most abundant grass was *Aristida junciformis*, which was more common in the non-encroached plains (34%) than in the streams (7%) and uplands (16%). The second most abundant grass was *Sporobolus africanus*, which revealed encounter rates of 18% in plains, 29% in streams and 9.6% in uplands. The main decreaser grass, *Themeda triandra*, only occurred in the plains, where it was only encountered at a rate of 1.9%. Overall, plant abundance was invariant among the plains, streams and uplands (Kruskal-Wallis *H* = 2.936, df = 2, *P* = 0.230). Similar abundance and species richness among site categories were also evident in the similar values of the Shannon-Weiner and Simpson diversity indices (Table 1).

**Table 1:** Mean (± SE) values of diversity and evenness among the non-encroached plains, streams and upland sites at Vuvu.

Sites	Diversity index		
	Shannon-Wiener (*Hʹ*)	Simpson (*D*)	Evenness (*E*)
Plains	1.40 ± 0.07a	0.32 ± 0.02a	0.40 ± 0.02a
Streams	1.41 ± 0.09a	0.32 ± 0.03a	0.40 ± 0.02a
Uplands	1.49 ± 0.06a	0.29 ± 0.02a	0.42 ± 0.02a

Values in a column with the same letter are not significantly different (*P* > 0.05).

### Relationship between *Leucosidea sericea* and herbaceous species composition

**Fig 1:**
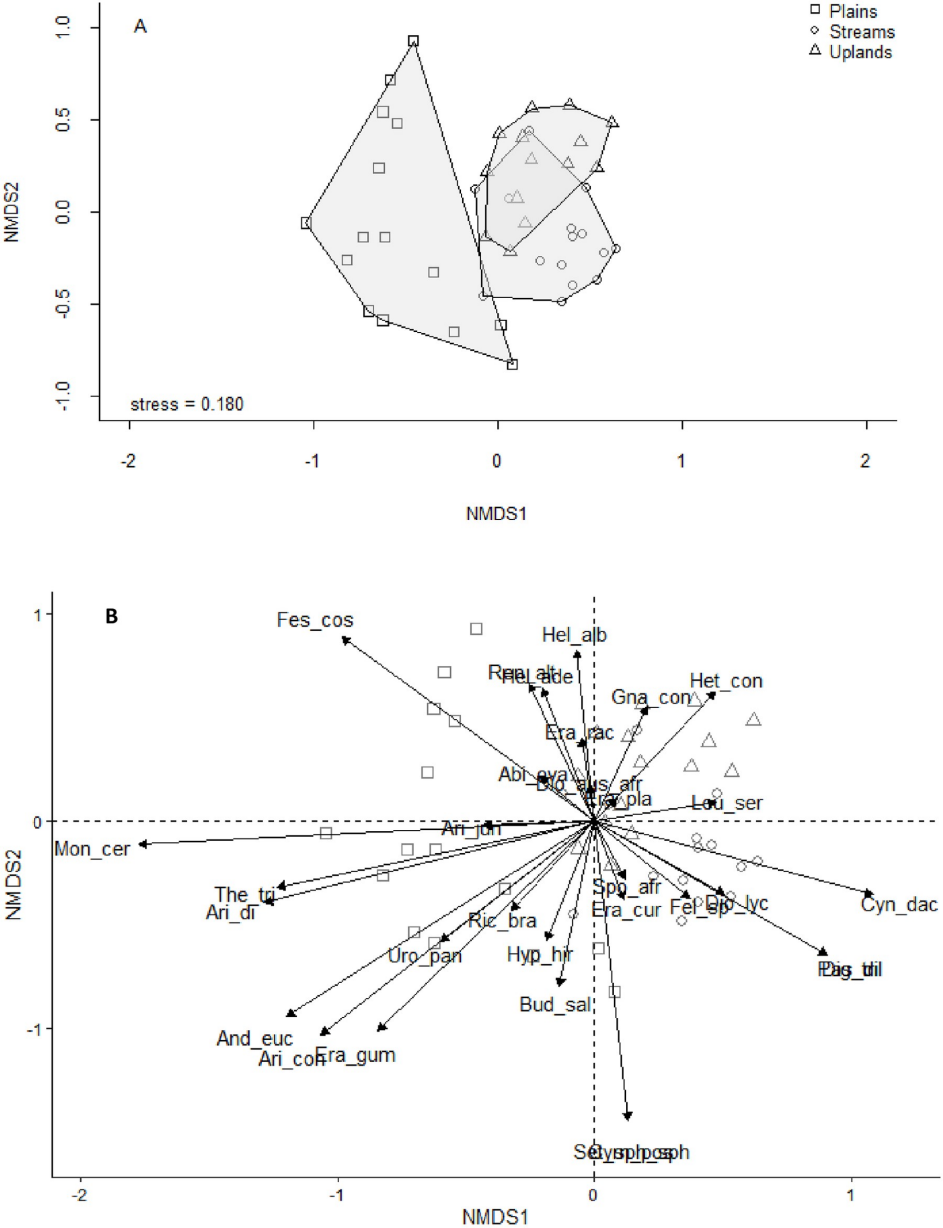
Non-metric multidimensional scaling of species composition based on Bray-Curtis dissimilarities between sites (n = 45 sites, stress = 0.18, *r*^2^ = 0.469, *P* < 0.001). A) Plant species composition in the topographical locations (plains, streams and uplands) at Vuvu, based on 15 sites in each case. B) Relationship between plant species and topographical locations. The longer the line the greater the influence of the species, with arrowheads indicating the direction of maximum change. Species are Fes_cos = *Festuca costata*, *Mon_cer = Monocymbium ceresiiforme*, *The_tri = Themeda triandra*, *Ari_dif = Aristida diffusa*, *And_euc = Andropogon eucomis*, *Aris_con_con = Aristida congesta* subsp. *congesta*, *Era_gum = Eragrostis gummiflua*, *Uro_pan = Urochloa panicoides*, *Ari_jun = Aristida junciformis*, *Ric_bra = Richardia brasiliensis*, *Hyp_hir = Hyparrhenia hirta*, *Ren_alt = Rendlia altera*, *Era_rac = Eragrostis racemosa*, *Het_con = Heteropogon contortus*, *Leu_ser = Leucosidea sericea*, *Spo_afr = Sporobolus africanus*, *Era_cur = Eragrostis curvula*, *Dio_lyc = Diospyros lycioides*, *Cyn_dac = Cynodon dactylon*, *Set_sph sph = Setaria sphacelata* subsp. *sphacelata*, *Fel_*sp. *= Felicia* sp., *Bud_sal = Buddleja salviifolia*.

A total of 17 grass species were recorded in the plains sites, compared to nine in streams and eight in uplands (Fig 1). For example, *Monocymbium ceresiiforme* and *Andropogon eucomis* occurred on the non-encroached plains but not in streams and uplands, while *Sporobolus africanus* and *Eragrostis plana* predominantly occurred in streams. The analysis of species composition across the study sites revealed a Bray-Curtis dissimilarity value of 66.73%. This level of dissimilarity suggests that each site had a different abundance of shared species. For instance, *Aristida junciformis* was more abundant in plains than in streams and uplands, whereas *S*. *africanus* showed a higher mean abundance in streams (S1 Table). Grass species that were less abundant among site categories included *Urochloa panicoides*, *Themeda triandra* and *Digitaria tricholaenoides* (S1 Table). The ANOSIM results indicated that there was a significant difference in species composition among the sites (R = 0.5, *P* <0.001; Fig 1).

### Effect of *Leucosidea sericea* trees on herbaceous basal cover

**Fig 2:**
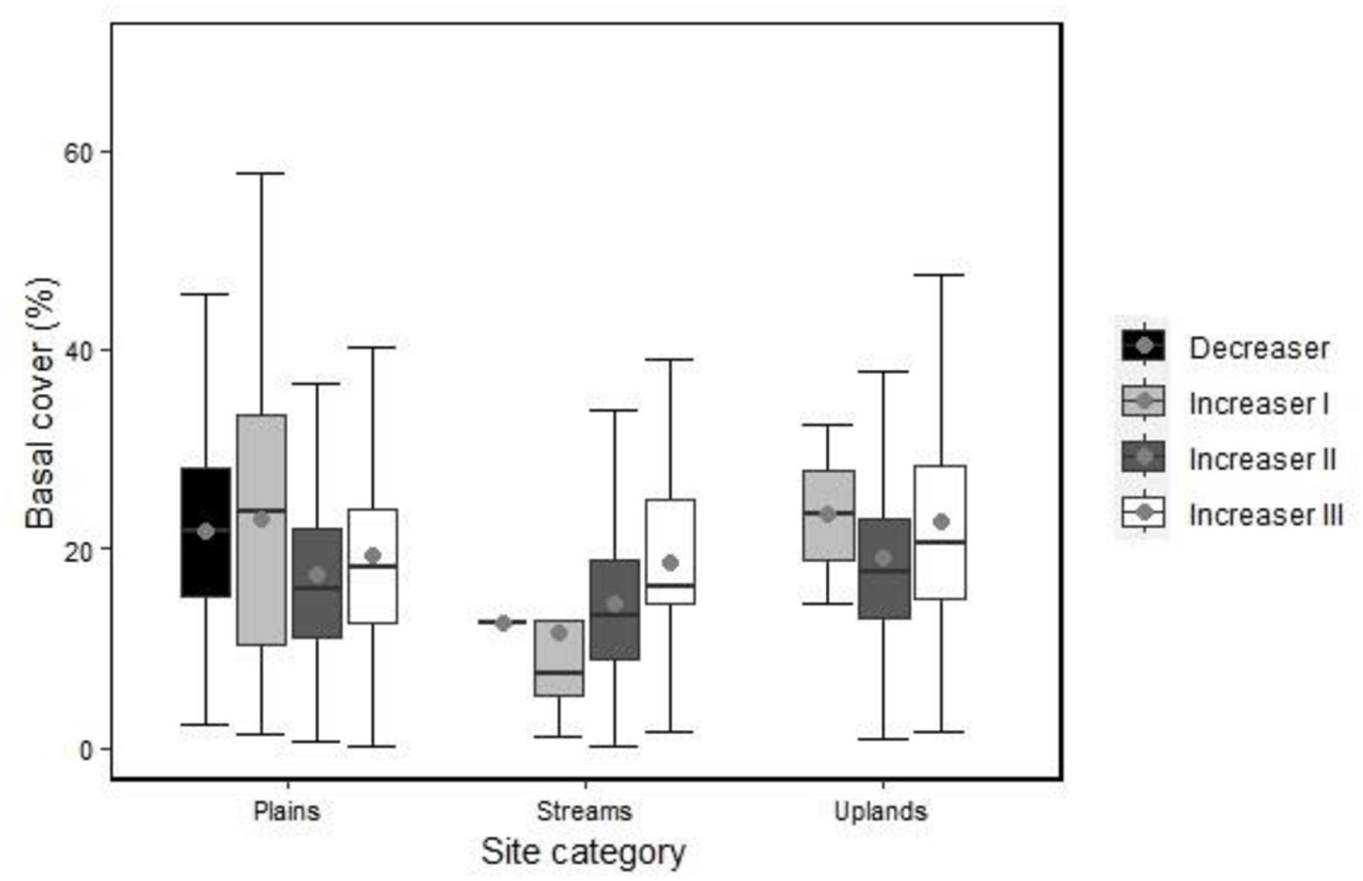
Percentage basal cover of ecological groups in the plains, streams and upland sites at Vuvu. Box plots show the medians (bold horizontal lines) with 25th and 75th percentiles (whiskers) and quartiles (interquartile range box and the medians).

There was an overall significant effect of location and ecological group on grass basal cover (likelihood ratio Wald’s chi-square = 105.92, df = 10, *P* < 0.001). Univariate tests showed that there were significant differences in basal cover among topographical locations and ecological groups (Table 2). In particular, highest values of basal cover were attained in the uplands and lowest in stream sites. Moreover, the basal cover of the increaser II grasses was significantly lower than that of the decreaser grasses while all other ecological groups had similar basal cover. However, there was no significant interaction between location and ecological groups (Table 2). Effects of *Leucosidea sericea* on ecological group basal cover in the plains, streams and uplands are shown in Fig 2. Basal cover on the non-encroached plain sites showed only marginal differences among the ecological groups. At stream sites, basal cover was particularly low for the increaser I group while it was comparable to that of the other locations for the increaser II and III groups. Decreaser species were notably absent from uplands. Of the four decreaser species, only *Themeda triandra* attained a significantly large rangeland condition score in the plains (S2 Table). The rangeland condition scores of increaser I species were invariant among locations, while that of increaser II species were significantly greater in uplands than plains for *E*. *curvula* and *H*. *contortus*. Among increaser III species, *Aristida junciformis* and *S*. *africanus* attained greatest rangeland condition scores in the uplands (S2 Table), which reflected results of the greater abundance of these species at uplands than streams and plain sites.

**Table 2:** Wald’s Chi-square and associated probabilities for the effect of ecological group and topographical location on grass basal cover.

Source of variation	df	Wald’s Chi-square	*P*
Ecological group (E)	3	26.473	**< 0.001**
Topographical location (S)	2	8.166	**0.017**
E × S	5	7.505	0.186

Significant p-values are in bold

### The impact of *Leucosidea sericea* on grazing capacity

**Fig 3:**
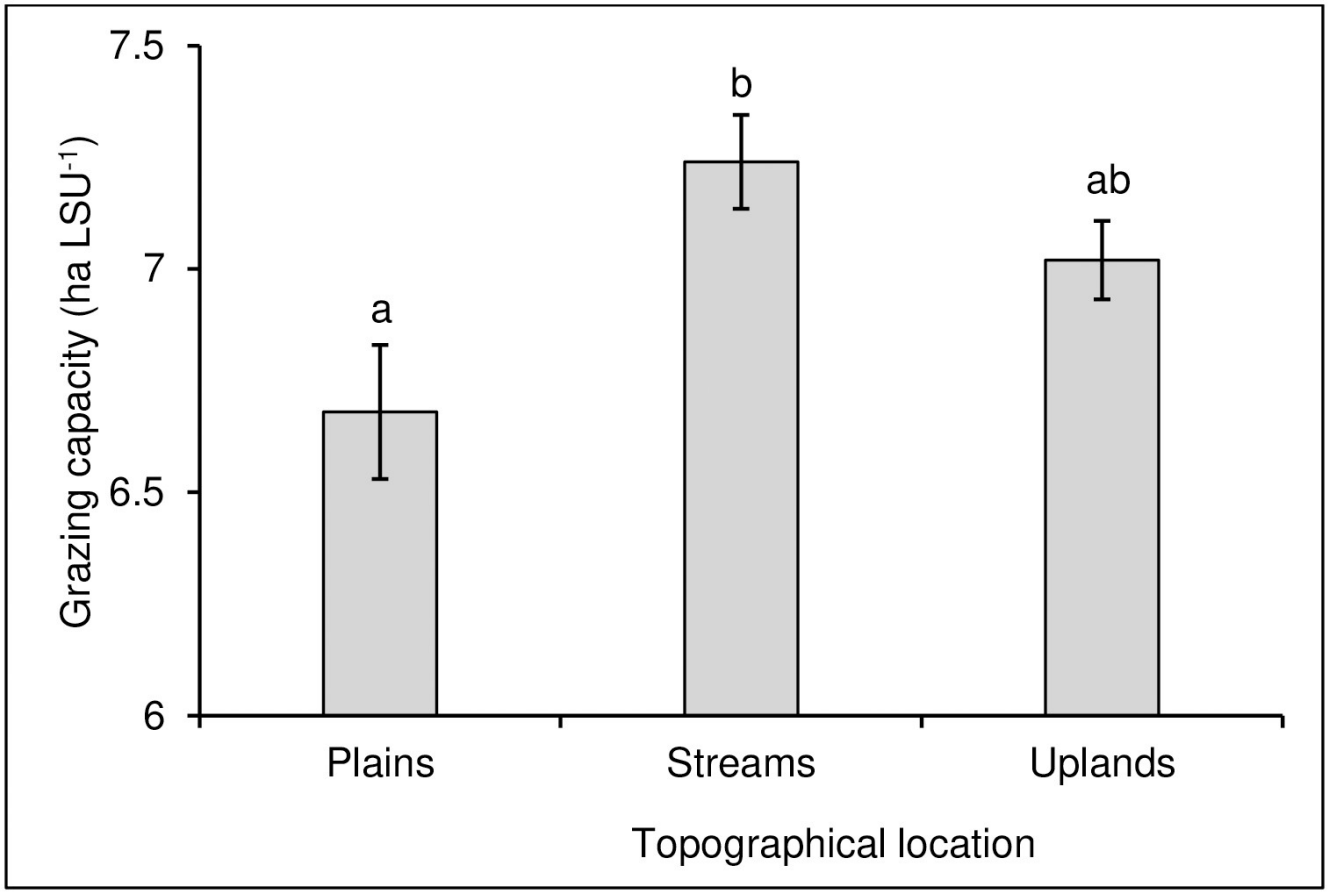
Grazing capacity at the plains, streams, and upland sites at Vuvu. Different superscript letters denote significant differences (*P* < 0.05) among topographical locations.

The grazing capacity of plain sites was approximately 8% lower than that of the stream sites, and the difference was significant (Fig 3). The grazing capacity of stream sites was similar to that of uplands and plain sites.

### Topographical locations and soil chemical properties

Soil physical and chemical property levels were similar among topographical locations of *L*. *sericea* (Pillai’s trace = 1.695, *F*_24, 4_ = 0.925, *P* = 0.612; Table 3). Soil was acidic across all topographical areas (range in pH: 4.4–4.6) and had low amounts of organic carbon (< 4%) and N (< 0.5%) (Table 3).

**Table 3:** Mean (±SE) amount of soil properties in the plains, streams and uplands at Vuvu, based on n = 5 samples in each case.

Soil property	Plains	Streams	Uplands
Sample density (g/mL)	1.01 (0.022)	1.02 (0.026)	1.02 (0.046)
Clay (%)*	24.60 (4.39)	22.40 (1.44)	22.20 (1.16)
Organic carbon (%)	3.52 (0.65)	3.08 (0.25)	2.84 (0.71)
Nitrogen (%)^#^	0.23 (0.059)	0.18 (0.019)	0.18 (0.053)
P (mg/kg)	4.40 (0.245)	4.0 (0.548)	5.0 (0.548)
K (mg/kg)*	270.8 (63.8)	248.2 (21.6)	212.6 (18.3)
Ca (mg/kg)	1144.8 (169.2)	1123.0 (225.9)	1020.6 (133.1)
Mg (mg/kg)	279.6 (54.6)	265.8 (47.3)	233.2 (35.9)
Mn (mg/kg)	19.20 (6.71)	11.80 (2.54)	15.40 (4.06)
Cu (mg/kg)*	1.88 (0.65)	1.40 (0.31)	1.76 (0.43)
Zn (mg/kg)	1.10 (0.38)	1.24 (0.37)	2.04 (0.89)
Exchangeable acidity (cmol/L)*	0.26 (0.11)	0.38 (0.20)	0.47 (0.17)
Acid saturation (%)^#^*	3.80 (1.88)	5.20 (3.12)	7.0 (3.42)
Total cations (cmol/L)^#^	8.97 (1.36)	9.30 (1.39)	8.02 (0.82)
pH (KCl)	4.49 (0.07)	4.61 (0.15)	4.40 (0.14)

^#^Parameter not used in the MANOVA described in the text because it was correlated with another variable

*Values calculated from data that were not normally distributed.

## Discussion

Species diversity did not respond to *L*. *sericea* densities on the various topographical locations (i.e. high density of *L*. *sericea* on uplands and streams, and low density of *L*. *sericea* on plains sites) (Table 1). This is contrary to what we expected that species diversity would be lower in streams and uplands because of high densities of *L*. *sericea* than in the plains without *L*. *sericea* plants. These expectations were based on high densities of *Leucosidea sericea* in streams and uplands, which could potentially outcompete other plants and reduce species diversity. This could significantly alter the habitat, making it less suitable for species that previously thrived there. Results from North America, Ethiopia and Limpopo Province of South Africa indicated that areas encroached by woody plants tend to have low plant species diversity while the non-encroached areas tended to have high species diversity [15, 24, 57]. The lack of significant differences in species diversity between the sites probably indicate that *L*. *sericea* encroachment has limited influence on herbaceous plants. [58] noted that species diversity needs to be carefully interpreted, because values of diversity indices might be similar among the sites, but the species composition may be significantly different.

Species composition is important for rangeland condition assessment as species may vary significantly in their resilience to grazing herbivores [59]. We found that the plains sites, devoid of *L*. *sericea* plants, had a greater variety of herbaceous species compared to the streams and upland sites, and this concurs with the prediction that high *L*. *sericea* densities would reduce species diversity on streams and uplands. Similarly, [24] indicated a decrease in herbaceous species diversity in the woody plant encroached grassland at Syferkuil Farm in Limpopo Province, South Africa. In the streams and uplands, some herbaceous species (e.g. *Eragrostis plana*, *E*. *curvula* and *Sporobolus africanus*) were dominant in the presence of high *L*. *sericea* densities. We are uncertain of the explanation for this, for these grasses are intolerant of shade, and increase in abundance in disturbed sites and overgrazed areas [46]. Although the plains had a high plant species diversity, most of the grasses occurring there (e.g. *Eragrostis gummiflua*, *Urochloa panicoides* and *Andropogon eucomus*) were less palatable as graze and commonly occur in disturbed habitats.

[60] reported a high abundance of unpalatable and less palatable herbaceous plants on sites that are heavily covered by *Senna obtusifolia* in regions of northern Ethiopia. The low occurrence of highly palatable species at our study site, such as *Themeda triandra*, is possibly a result of rangeland degradation arising from disturbances such as previous cultivation and high densities of woody plants, or woody plant encroachment [61]. In the 1950s and 1960s, the government’s Betterment Programme to reduce soil erosion, promote environmental conservation, and improve agricultural production was being implemented through relocation of homesteads, separation of crop fields from grazing camps through fencing and establishment of seasonal grazing areas [62]. These developments may have initiated the increase in density of *L*. *sericea*. Some of the repercussions of field abandonment include the replacement of perennial grasses with shrubs, loss of species diversity that may compromise soil biophysical qualities, and increased rates of soil erosion [63–65], all of which may be exacerbated by overgrazing. The increased density of *L*. *sericea* may have been driven by the relocation of people and other factors such as rainfall amount, change in grazing pattern and occurrence of fires of low intensity. Increased rainfall can significantly affect the growth and spread of woody plants [66–68]. Higher rainfall may help *L*. *sericea* to spread if it thrives in environments of high moisture amounts, while tree seedlings establishing in overgrazed sites may develop without fire, thereby increasing woody plant density [12, 69–71].

Based on the basal cover of ecological groups, it was found that the grassy plains had high values compared to streams and uplands; these results could be attributed to the presence of *L*. *sericea* in the latter. However, high densities of *L*. *sericea* is not the only problem in the area, for veld fires may have contributed to the reduction of basal cover, especially in Vuvu, with its history of frequent, anthropogenically-derived fires. A medium altitude (ca. 1450 masl) mesic grassland at Thaba Nchu in the Free State also showed decreased grass basal cover with fire [72]. Nevertheless, we are uncertain about how fire is managed in Vuvu. It is likely that the wooded streams and uplands are burnt more frequently than the plains, such as for subsistence hunting.

Traditional range management practices such as lighting fires to promote nitrogen-rich grass regrowth may be harmful to fire-sensitive species whose abundance would decline with frequent burning thereby making rangelands less valuable and less diverse [73, 74]. Nonetheless, large frequent fires allow for sparse distribution of grazing on the regrowth so that there is less concentration of animals in any one area [75]. In Vuvu, fires are unlikely to be large owing to the high grazer densities, while the *L*. *sericea* encroached sites would also have lower herbaceous fuel loads.

The findings on rangeland condition showed that the plains, streams and uplands were dominated by increaser II and increaser III species, which are less palatable to livestock. These results were contrary to what we expected in the plains but concurs with expectations at streams and uplands. We expected that less steep plains would have more palatable species (decreaser and increaser I) since they were not encroached by *L*. *sericea*, but the main grasses identified from the non-encroached plains, streams and uplands were *Aristida junciformis* and *Sporobolus africanus*. The presence of these grasses indicates poor rangeland condition, which suggests that rangeland management needs to increase the proportion of palatable grasses for livestock.

Over longer time scales, unsustainable grazing can affect competition between plant species, initially reducing perennial grass cover in favour of less palatable annual grasses and ultimately replacing grasses with shrubs [76, 77]. Sheep are a preferred type of livestock in the area [27], but are potentially harmful to the rangeland due to their selective grazing and ability to graze plants low to the ground level, consequently destroying plant growth portions [78]. Furthermore, horses and donkeys graze down plants near to the ground surface, which can be highly harmful to the plants if not controlled [47]. Thus, uncontrolled grazing might contribute to the deterioration of rangeland in the area.

We expected greater grazing capacity in the plains than in streams and uplands, and we further expected that the plains would have high abundance and cover of palatable species (decreaser and increaser I). Our overall results demonstrated low grazing capacity with values ranging from 6.7 to 7.2 ha LSU^-1^, which could be attributed to the low abundance of decreaser and increaser I species. At Stutterheim in the Eastern Cape Province of South Africa, a rangeland managed by communal and commercial farmers recorded grazing capacity of 8 ha LSU^-1^ in highly encroached sites to 2 ha LSU^-1^ in non-encroached sites [79]. At Matatiele, also in the Eastern Cape, [80] reported greater grazing capacity (range 1.07 ha LSU^-1^ to 2.15 ha LSU^-1^) in three communal areas where coordinated grazing management in the form of rotational grazing was taking place. The low grazing capacity obtained from this study indicates that the rangeland may be degraded severely soon if interventions to arrest the decline are not implemented. The amount of plant material generated per hectare may be heavily influenced by rainfall. Rainfall has a direct impact on the amount of fodder produced for herbivore consumption [81]. Depending on the amount of rainfall and other things being equal, the higher the rainfall, the greater the grazing capacity.

Soil nutrient status showed similar patterns among the topographical locations and consequently the densities of *L*. *sericea*. Soil organic carbon was similar among the plains, streams, and uplands. In contrast, the Stutterheim study showed that encroached sites had high levels of soil C [79]. In addition, [82] indicated a strong negative effect of *Acacia mearnsii* encroachment on soil C in the Motseng area of Matatiele in the Eastern Cape. The low amounts of soil C could be attributed to grazing and burning as these are major controls of carbon input quantity and quality in grasslands [82]. The soil carbon may initially increase with woody plant density, but then declines when woody plant density becomes high, which also inhibits understory grass growth [76, 83]. This suggests that most soil C is derived from herbaceous plants such as grasses, which declines in abundance with increased density of woody plants. Also, over-utilisation of rangelands through heavy grazing may potentially reduce amounts of soil C [84]. The amount of soil N did not differ among the plains, streams and upland sites. This is contrary to findings by [23], who reported significantly high concentrations of soil N in sites of high tree density compared to open areas in a semi-arid savanna at Windhoek, Namibia.

Available phosphorus was also similar among the topographic locations. The literature indicates mixed results of soil P content under various densities of woody plants. Similar to the findings of this study, [23] reported similar amounts of soil P under high and moderate density of woody plants in Namibia. [85] also found similar amounts of soil P in *Acacia melllifera* encroached and open grassland sites of Pomfret in the North-West of South Africa. However, [86] reported different results in semi-arid rangeland at Pniel Estate and Koopmansfontein near Kimberly in the Northern Cape, South Africa, where higher P concentrations were recorded under *A*. *mellifera* than in open grasslands. We found similarly acidic levels of soil pH in the plains, streams and uplands sites. Acidic soil may be attributed to high amounts of rainfall where leaching could possibly result in low pH. Additionally, land management such as frequent burning and high intensity grazing may influence soil chemical properties. Overall, the amounts of soil nutrients in the sites of different densities of *L*. *sericea* showed that soils were nutrient-poor. Moreover, the patterns of plant species richness and species diversity also showed a rangeland ecosystem that is species-poor. Although the rangeland occurs on the boundaries of a centre of plant endemism, many of the endemic species indicated in [32] were not encountered at Vuvu. In addition, a comparison of grassland species diversity in restored and intact coastal grassland in KwaZulu-Natal showed that plant diversity was much greater in intact- (221 species) than restored grassland (144 species) [87], which reiterates that the Vuvu rangeland is depauperate in plant species richness. However, the grasslands in the [87] study occur in a biodiversity hotspot.

This study contributes to a broader understanding of the dynamics of *L*. *sericea* encroachment in grassland ecosystems. The study highlights the significant role of *L*. *sericea* in altering the grazing capacity of high-altitude rangelands, which has implications for future research and rangeland management. By demonstrating the potential impact of *L*. *sericea*, our findings suggest several pathways for further research. We recommend that future research identify and elucidate mechanisms underlying the interactions between *L*. *sericea* and herbaceous plants, considering factors such as competition for resources, fire regimes, and climate change impacts. This could pave the way for building ecological models and predictive frameworks to inform about ecosystem responses to environmental change [see 88]. Machine learning algorithms such as Random Forest regression [89] can elucidate the relative importance of biotic and abiotic factors in driving grazing capacity. We recommend use of these strategies to enhance the accuracy and reliability of predictions about rangeland functioning. Rangeland management should also focus on ecological restoration beyond concerns about controlling the density of *L*. *sericea*. We recommend enrichment seeding of decreaser grasses, such as *Digitaria tricholaenoides*, *Monocymbium ceresiiforme*, *Setaria sphacelata sphacelata*, and *Themeda triandra*, all of which already occur in the area, albeit in low amounts. Moreover, integrating the insights from this study in grassland management can enhance the effectiveness of rangeland conservation. For instance, adopting a holistic approach that considers both ecological and socio-economic factors can lead to more sustainable rangeland management practices [90]. By engaging local communities and other stakeholders, management strategies can be tailored to address specific challenges while promoting the long-term resilience of grassland ecosystems. We argue that local communities be put at the centre of rangeland management, coupled with providing environmental education on sustainable grazing methods, and establishing participatory rangeland condition assessment and monitoring approaches led by the community [91]. This allows integration of approaches from traditional knowledge of the community and scientific knowledge of researchers in managing rangelands [92]. Finally, our findings underscore the importance of incorporating ecological research into policy and decision-making processes related to mitigating overutilisation of communally-managed, finite resources. By recognising the ecological significance of grasslands and the threats posed by *L*. *sericea* encroachment, policymakers can develop targeted interventions and incentives to support sustainable land management practices.

### Limitations of the research

Although this study offers insights into the effects of *L*. *sericea* on grazing capacity and rangeland condition, we acknowledge limitations. Firstly, the study was conducted in a particular high-altitude communal rangeland in Vuvu. Findings of this research may lack generalisation to other rangelands. Secondly, field sampling was undertaken in one growing season, which limited the study from capturing long-term patterns essential for assessing the sustained impact of *L*. *sericea* on grazing capacity, rangeland condition and soil characteristics. Finally, soil samples were taken to a depth of 5 cm. The limited depth of sampling may be inadequate to show the distribution of nutrients in landscapes of various topography, vegetation types and land use [93].

## Conclusion

The rangeland condition at Vuvu is poor. The hypothesis that *L*. *sericea* has negative impacts on streams and uplands compared to plains was not supported. Findings of the study indicated lower abundance and number of decreaser species in all the sites, and a greater presence of increaser II (less palatable) species. This implies that both the quantity and quality of forage has declined. The poor conditions cannot be attributed solely to greater densities of *L*. *sericea* because the plains were also characterised by poor rangeland conditions. Rangeland condition change in Vuvu suggests that the extent and severity of human impacts on herbaceous plant species have increased significantly over time, possibly through overgrazing and poor fire management. Given these adverse effects on rangeland condition leading to degradation, urgent control measures together with ecological restoration may be required to improve the rangeland. There is a need to increase the abundance of palatable grasses such as decreaser species with high grazing value to improve rangeland condition. This can be achieved through various mechanisms including a better distribution of livestock such as through rotational grazing and introduction of a prescribed burning regime.

## Supporting information

S1 Table Contribution of plant species to compositional dissimilarities on the plains, streams and uplands, based on 15 sites in each case. (DOCX)

S2 Table Mean (±1SE) rangeland condition scores for plants on sites located in non-encroached plains, streams, and uplands at Vuvu. Means in the same row with different letters are significantly different (*P* < 0.05). Significant *p*-values are indicated in bold. In all cases, n = 15.
(DOCX)

S1 Fig Frequency of occurrence of *L*. *sericea* trees of different height classes at stream and upland sites. (DOCX)
