# Supplementary material for: Increasing densities of Leucosidea sericea have minimal effects on grazing capacity and soil characteristics of a high-altitude communal rangeland at Vuvu, South Africa
Source: PLoS One. 2024 Sep 6;19(9):e0308472. doi: 10.1371/journal.pone.0308472 (PMC11379305; doi:10.1371/journal.pone.0308472)
Supplement: S1 Table — (DOCX) [file pone.0308472.s001.docx]

S1 Table. Contribution of plant species to compositional dissimilarities on the plains, streams and uplands, based on 15 sites in each case.

| Species | Average dissimilarity | Contribution % | Cumulative % | Plains | Streams | Uplands |
| --- | --- | --- | --- | --- | --- | --- |
| *Leucosidea sericea* | 14.44 | 21.33 | 21.33 | 0 | 16.1 | 17.30 |
| *Aristida junciformis* | 11.55 | 17.07 | 38.40 | 15.90 | 3.60 | 7.40 |
| *Sporobolus africanus* | 9.86 | 14.57 | 52.97 | 8.33 | 14.1 | 4.53 |
| *Heteropogon contortus* | 5.65 | 8.34 | 61.32 | 0.13 | 1.47 | 7.33 |
| *Eragrostis plana* | 5.20 | 7.69 | 69.00 | 4.00 | 5.00 | 3.53 |
| *E. curvula* | 4.01 | 5.91 | 74.91 | 2.80 | 2.73 | 2.60 |
| *Festuca costata* | 3.49 | 5.16 | 80.08 | 4.87 | 0 | 0 |
| *Rendlia altera* | 2.3 | 3.30 | 83.48 | 1.87 | 0.27 | 1.87 |
| *E. racemosa* | 1.24 | 1.80 | 85.30 | 0.67 | 0.13 | 1.33 |
| *Helichrysum albirosulatum* | 1.09 | 1.60 | 86.90 | 0.53 | 0.80 | 0.33 |
| *Richardia brasiliensis* | 0.90 | 1.47 | 88.37 | 0.93 | 0.33 | 0.33 |
| *Hyparrhenia hirta* | 0.80 | 1.33 | 89.70 | 1.00 | 0.33 | 0.13 |
| *Felicia* sp*.* | 0.85 | 1.26 | 90.96 | 0 | 1.27 | 0 |
| *Monocymbium ceresiiforme* | 0.70 | 1.18 | 92.14 | 1.13 | 0 | 0 |
| *Diospyros lycioides* | 0.67 | 0.98 | 93.12 | 0 | 0.93 | 0.07 |
| *Themeda triandra* | 0.61 | 0.90 | 94.03 | 0.87 | 0 | 0 |
| *Andropogon eucomis* | 0.55 | 0.81 | 94.83 | 0.80 | 0 | 0 |
| *Gnaphalium confine* | 0.49 | 0.74 | 95.57 | 0.20 | 0.27 | 0.33 |
| *Helichrysum adenocarpum* | 0.48 | 0.71 | 96.28 | 0.40 | 0.27 | 0 |
| *Cymbopogon pospichilli* | 0.37 | 0.55 | 96.83 | 0.53 | 0 | 0 |
| *Urochloa panicoides* | 0.36 | 0.54 | 97.36 | 0.53 | 0 | 0 |
| *E. gummiflua* | 0.28 | 0.41 | 97.78 | 0.40 | 0 | 0 |
| *Paspalum dilatatum* | 0.28 | 0.41 | 98.18 | 0 | 0.40 | 0 |
| *Cynodon dactylon* | 0.25 | 0.37 | 98.55 | 0 | 0.33 | 0 |
| *Aristida diffusa* | 0.24 | 0.35 | 98.9 | 0.33 | 0 | 0 |
| *Setaria sphacelata sphacelata* | 0.23 | 0.34 | 99.24 | 033 | 0 | 0 |
| *Aristida congesta congesta* | 0.14 | 0.21 | 99.45 | 0.20 | 0 | 0 |
| *Acacia mearnsii* | 0.09 | 0.14 | 99.59 | 0 | 0.13 | 0 |
| *Diospyros austro-africana* | 0.09 | 0.14 | 99.73 | 0 | 0 | 0.13 |
| *Abildgaardia ovata* | 0.09 | 0.14 | 99.87 | 0 | 0 | 0.13 |
| *Aloe_*sp*.* | 0.05 | 0.07 | 99.94 | 0 | 0.07 | 0 |
| *Digitaria* *tricholaenoides* | 0.04 | 0.06 | 100.00 | 0 | 0.07 | 0 |
