# Supplementary material for: Increasing densities of Leucosidea sericea have minimal effects on grazing capacity and soil characteristics of a high-altitude communal rangeland at Vuvu, South Africa
Source: PLoS One. 2024 Sep 6;19(9):e0308472. doi: 10.1371/journal.pone.0308472 (PMC11379305; doi:10.1371/journal.pone.0308472)
Supplement: S2 Table — Means in the same row with different letters are significantly different (P < 0.05). Significant p-values are indicated in bold. In all cases, n = 15. (DOCX) [file pone.0308472.s002.docx]

S2 Table. Mean (±1SE) rangeland condition scores for plants on sites located in non-encroached plains, streams, and uplands at Vuvu. Means in the same row with different letters are significantly different (*P* < 0.05). Significant *p*-values are indicated in bold. In all cases, n = 15.

| **Species** | **Plains** | **Streams** | **Uplands** | ***F*** | ***P*** |
| --- | --- | --- | --- | --- | --- |
| **Decreasers** |  |  |  |  |  |
| *Digitaria tricholaenoides* | 0.00 ± 0.00a | 0.67 ± 2.58a | 0.00 ± 0.00a | 1.00 | 0.37 |
| *Monocymbium ceresiiforme* | 11.33 ± 11.33a | 0.00 ± 0.00a | 0.00 ± 0.00a | 1.00 | 0.37 |
| *Setaria sphacelata sphacelata* | 3.33 ± 3.33a | 0.00 ± 0.00a | 0.00 ± 0.00a | 1.00 | 0.37 |
| *Themeda triandra* | 8.67 ± 4.67a | 0.00 ± 0.00b | 0.00 ± 0.00b | 3.45 | **0.04** |
| **Increaser I** |  |  |  |  |  |
| *Cymbopogon pospichilli* | 3.73 ± 3.73a | 0.00 ± 0.00a | 0.00 ± 0.00a | 1.00 | 0.37 |
| *Hyparrhenia hirta* | 4.0 ± 1.70a | 1.33 ± 0.50a | 0.53 ± 0.53a | 2.88 | 0.06 |
| **Increaser II** |  |  |  |  |  |
| *Andropogon eucomis* | 3.20 ± 3.20a | 0.00 ± 0.00a | 0.00 ± 0.00a | 1.00 | 0.37 |
| *Aristida congesta congesta* | 0.80 ± 0.80a | 0.00 ± 0.00a | 0.00 ± 0.00a | 1.00 | 0.37 |
| *Cynodon dactylon* | 0.00 ± 0.00a | 1.33 ± 1.33a | 0.00 ± 0.00a | 1.00 | 0.37 |
| *Eragrostis curvula* | 11.20 ± 4.54a | 10.93 ± 4.23a | 10.40 ± 3.82a | 0.01 | 0.99 |
| *Eragrostis gummiflua* | 1.60 ± 0.94a | 0.00 ± 0.00a | 0.00 ± 0.00a | 2.91 | 0.06 |
| *Eragrostis plana* | 16.00 ± 5.09a | 20.00 ± 6.37a | 14.13 ± 3.84a | 0.33 | 0.71 |
| *Eragrostis racemosa* | 2.67 ± 1.28b | 0.53 ± 0.36b | 5.33 ± 1.86a | 3.31 | **0.04** |
| *Heteropogon contortus* | 0.13 ± 0.13b | 1.47 ± 0.87a | 7.33 ± 2.14a | 8.25 | **0.01** |
| *Urochloa panicoides* | 2.13 ± 2.13a | 0.00 ± 0.00a | 0.00 ± 0.00a | 1.00 | 0.37 |
| **Increaser III** |  |  |  |  |  |
| *Aristida diffusa* | 0.33 ± 0.27a | 0.00 ± 0.00a | 0.00 ± 0.00a | 1.52 | 0.23 |
| *Aristida junciformis* | 15.93 ± 2.75a | 3.60 ± 1.38a | 7.40 ± 1.75b | 9.55 | **0.01** |
| *Paspalum dilatatum* | 0.00 ± 0.00a | 0.40 ± 0.40a | 0.13 ± 0.13a | 1.00 | 0.37 |
| *Rendlia altera* | 1.87 ± 0.75a | 0.27 ± 0.27a | 0.87 ± 0.63a | 2.48 | 0.09 |
| *Sporobolus africanus* | 8.93 ± 2.31b | 14.13 ± 2.20b | 4.53 ± 1.26a | 5.10 | **0.01** |
|  |  |  |  |  |  |
