# Supplementary material for: Increasing densities of Leucosidea sericea have minimal effects on grazing capacity and soil characteristics of a high-altitude communal rangeland at Vuvu, South Africa
Source: PLoS One. 2024 Sep 6;19(9):e0308472. doi: 10.1371/journal.pone.0308472 (PMC11379305; doi:10.1371/journal.pone.0308472)
Supplement: S1 Fig — (DOCX) [file pone.0308472.s003.docx]

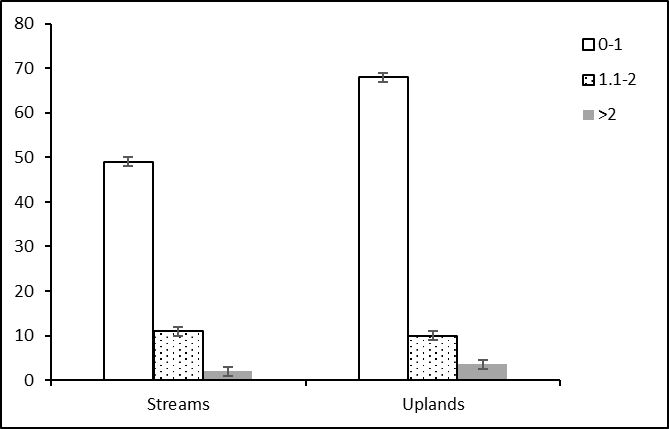


S1 Fig. Frequency of occurrence of *L. sericea* trees of different height classes at stream and upland sites.
